# Supplementary material for: Multi‐dataset identification of innovative feature genes and molecular mechanisms in keratoconus
Source: J Cell Mol Med. 2024 Sep 19;28(18):e70079. doi: 10.1111/jcmm.70079 (PMC11412914; doi:10.1111/jcmm.70079)
Supplement: Supplementary file 2 — Material S2. [file JCMM-28-e70079-s002.zip › 7.GSEA analysis/3.DBNDD1/KEGG_GLYOXYLATE_AND_DICARBOXYLATE_METABOLISM.html]

Details for gene set KEGG\_GLYOXYLATE\_AND\_DICARBOXYLATE\_METABOLISM[GSEA]

|  || Dataset | DBNDD1.group.cls#h\_versus\_l |
| Phenotype | group.cls#h\_versus\_l |
| Upregulated in class | h |
| GeneSet | KEGG\_GLYOXYLATE\_AND\_DICARBOXYLATE\_METABOLISM |
| Enrichment Score (ES) | 0.4877708 |
| Normalized Enrichment Score (NES) | 1.3615245 |
| Nominal p-value | 0.13241106 |
| FDR q-value | 0.6458148 |
| FWER p-Value | 0.92 |
Table: GSEA Results Summary

  

Fig 1: Enrichment plot: KEGG\_GLYOXYLATE\_AND\_DICARBOXYLATE\_METABOLISM      
 Profile of the Running ES Score & Positions of GeneSet Members on the Rank Ordered List

  

| SYMBOL | TITLE | RANK IN GENE LIST | RANK METRIC SCORE | RUNNING ES | CORE ENRICHMENT || 1 | HAO1 | na | 540 | 0.440 | 0.2970 | Yes |
| 2 | HAO2 | na | 4354 | 0.202 | 0.3304 | Yes |
| 3 | GLYCTK | na | 5037 | 0.179 | 0.4381 | Yes |
| 4 | AFMID | na | 6612 | 0.134 | 0.4878 | Yes |
| 5 | GRHPR | na | 10424 | 0.065 | 0.4245 | No |
| 6 | MDH2 | na | 13081 | 0.038 | 0.3747 | No |
| 7 | PGP | na | 13877 | 0.031 | 0.3737 | No |
| 8 | MTHFD2 | na | 16631 | 0.012 | 0.3028 | No |
| 9 | ACO2 | na | 18891 | -0.004 | 0.2403 | No |
| 10 | CS | na | 19772 | -0.011 | 0.2228 | No |
| 11 | MDH1 | na | 19861 | -0.012 | 0.2286 | No |
| 12 | MTHFD2L | na | 20844 | -0.021 | 0.2149 | No |
| 13 | ACO1 | na | 22387 | -0.036 | 0.1961 | No |
| 14 | MTHFD1 | na | 23479 | -0.049 | 0.1996 | No |
| 15 | HYI | na | 24778 | -0.068 | 0.2102 | No |
| 16 | MTHFD1L | na | 26992 | -0.108 | 0.2229 | No |
Table: GSEA details [plain text format]

  

Fig 2: KEGG\_GLYOXYLATE\_AND\_DICARBOXYLATE\_METABOLISM      
 Blue-Pink O' Gram in the Space of the Analyzed GeneSet

  

Fig 3: KEGG\_GLYOXYLATE\_AND\_DICARBOXYLATE\_METABOLISM: Random ES distribution      
 Gene set null distribution of ES for **KEGG\_GLYOXYLATE\_AND\_DICARBOXYLATE\_METABOLISM**
